# Levels of Essential Elements in Different Medicinal Plants Determined by Using Inductively Coupled Plasma Mass Spectrometry

**DOI:** 10.1155/2018/7264892

**Published:** 2018-03-20

**Authors:** Eid I. Brima

**Affiliations:** Department of Chemistry, Faculty of Science, King Khalid University, P.O. Box 9004, Abha 61413, Saudi Arabia

## Abstract

The objective of this study was to investigate the content of essential elements in medicinal plants in the Kingdom of Saudi Arabia (KSA). Five different medical plants (mahareeb *(Cymbopogon schoenanthus)*, sheeh *(Artemisia vulgaris)*, harjal *(Cynanchum argel delile)*, nabipoot *(Equisetum arvense)*, and cafmariam *(Vitex agnus-castus)*) were collected from Madina city in the KSA. Five elements Fe, Mn, Zn, Cu, and Se were determined by using inductively coupled plasma mass spectrometry (ICP-MS). Fe levels were the highest and Se levels were the lowest in all plants. The range levels of all elements in all plants were as follows: Fe 193.4–1757.9, Mn 23.6–143.7, Zn 15.4–32.7, Se 0.13–0.92, and Cu 11.3–21.8 *µ*g/g. Intakes of essential elements from the medical plants in infusion were calculated: Fe 4.6–13.4, Mn 6.7–123.2, Zn 7.0–42.7, Se 0.14–1.5, and Cu 1.5–5.0 *µ*g/dose. The calculated intakes of essential elements for all plants did not exceed the daily intake set by the World Health Organization (WHO) and European Food Safety Authority (EFSA). These medicinal plants may be useful sources of essential elements, which are vital for health.

## 1. Introduction

Medicinal plants are used by millions of people around the world. The usage of medicinal plants was mainly in primary healthcare by most of the users [1]. This study focused on some of medicinal plants used in the Kingdom of Saudi Arabia (KSA). Based on personnel communications from users and retailers, the investigated plants are used as follows: sheeh is used for the treatment of diseases related to respiratory and reproductive systems; mahareeb is used as a diuretic; harjal is used to alleviate gastrointestinal pain; cafmariam is used to regulate menstruation, and it is also used in dystocia and its soaked infusion to accelerate delivery; and nabipoot is used to ease pain associated with obstetrics. Some of micronutrients (Zn, Mn, Fe, Cu, and Se) were the core of this study to be investigated in the abovementioned medicinal plants. Different elements are classified as macronutrients or micronutrients based on their amount needed for human body. Consequently, microminerals are needed in less amount than macroelements such as K, Na, Ca, P, and Mg [2].

Copper is found in various human transcription factors and enzymes and is considered as an important essential trace element. However, excessive intake of copper can lead to liver damage and Wilson's disease. Wilson's disease results from accumulation of free copper in the liver, brain, and kidney [3]. Deficiency of copper can have adverse health effects, including aneurysms, blood vessel damage, hernias, and nosebleeds and can also affect the movement of nutrients through cell walls [4].

Iron is well established as an essential element and is a component of haemoglobin and is present in several human enzymes. Iron is present in high levels in red blood cells and muscle tissue. High doses of iron can cause hepatotoxicity. Acute liver failure has been reported after an overdose of ferrous sulphate in a young adult [5]. Iron deficiency leads to anaemia, which is the most well-known nutritional deficiency disorder [6].

Manganese is an important element and is a constituent of metalloenzymes that oxidize cholesterol and fatty acids [7]. A deficiency in manganese can cause bleeding disorders, while excessive amounts can cause speech disorders, leg cramps, and encephalitis [8].

Selenium is an antioxidant and is involved in the biosynthesis of thyroid hormones. Selenium plays an important role in the immune system and can help suppress the progression of HIV to AIDS [9]. Intake of excess selenium is associated with Keshan disease, which causes an enlarged heart, cardiac arrhythmias, and congestive heart failure [10, 11]. Selenium deficiency affects the function of selenoproteins and can cause damage to tissues and organs [12]. In addition, selenium deficiency can cause muscle weakness, skeletal myopathy, cardiomyopathy, and red blood cell macrocytosis [13].

Zinc is an essential element, and cellular zinc promotes homeostatic control to avoid accumulation of surplus zinc. Exposure to excessive zinc results in copper deficiency and cell apoptosis. In addition, zinc deficiency has been linked to a suppressed immune response [14]. Zinc and copper are considered important for metabolizing glucose and lowering cholesterol [14, 15].

Subramanian et al. [16] determined the Fe, Zn, Cu, and Mn contents in two tropical plants. They found the average concentrations (mg/kg) for these elements in the leaves of *Syzygium caryophyllatum* and *Syzygium densiflorum* were Fe (99.3, 93.8), Zn (17.3, 17.4), Cu (45.5, 64.9) and Mn (47.3, 31.8), respectively. The study concluded that these two plants were rich in some trace elements which are necessary for human health.

Different essential elements with levels (mg/kg), including Fe (54.2–231), Zn (4.7–19), Cu (3.2–10.5), and Mn (8.8–490), were previously determined in herbs and spices from Saudi Arabia [17], and no risk associated with these elements was identified even with a daily intake of 10 g of the investigated spices.

Essential elements including Fe, Zn, Mn, and Cu were determined in six medicinal plants (*Zizyphus jujube, Eugenia jambolana, Coccinia indica, Citrus acida, Ocimum sanctum*, and *Trigonella foenumgraecum*) from India. The concentrations (mg/kg) were in the following ranges: Fe 123–760, Zn 20.4–620, Mn 17.5–426, and Cu 0.4–35.5. The study concluded that these plants were rich in one or more of the investigated elements [18]. Essential elements including Fe, Se, Zn, Cu, and Mn were determined in six medicinal plants (*Emblica officinalis*, *Catharanthus roseus*, *Azadirachta indica*, *Solanum anguivi*, *Artemisia nilargirica*, and *Elsholtzia communis*) from India. The levels (mg/kg) of the elements in the plants were in the following ranges: Mn 31.3–183.7, Fe 311.1–820.4, Cu 3.7–30.1, Zn 23.3–62.8, and Se 0.0–0.5. They stated that trace elements in medicinal plants play an important role in treatment of disease [19].

The aim of this study was to assess the levels of essential elements in local medicinal plants from the KSA using ICP-MS. This study is necessary, because the studied plants are potential source of micronutrient elements such as Fe, Cu, Zn, Mn, and Se, which are considered as anti-oxidant elements as well. These medicinal plants could make a contribution to intake of essential elements based on their use to treat different diseases. The second purpose was also to calculate intake of each element per dose of each plant.

## 2. Materials and Methods

Toxic elements (Al, Pb, As, and Cd) were already determined in the five medicinal plants mahareeb *(Cymbopogon schoenanthus)*, sheeh *(Artemisia vulgaris)*, harjal *(Cynanchum argel delile)*, nabipoot *(Equisetum arvense)*, and cafmariam *(Vitex agnus-castus)*. Therefore, all subsections related to sample collection and preparation, sample digestion, elemental measurements by using ICP-MS, chemicals reagents, and analytical method were the same as the previous study [20].

### 2.1. Limits of Detection (LODs) and Limit of Quantification (LOQs)

The LODs were calculated after measuring 1% HNO_3_ 10 times. Limits of detection and quantification were calculated  by the following formulas:  LOD = 3 × SD and LOQ = 10 × SD.

### 2.2. Quality Control

For quality control, continuing calibration verification (CCV) analysis was used for each run. The CCV was performed by testing 20 *μ*g/L of a mixed standard of all measured elements after each set of five samples. A sample was spiked with mixed standards (20 *μ*g/L) of analysed elements, and the recovery was calculated for each element.

### 2.3. Statistical Analysis

Statistical analysis was performed on all plant samples using the two-sample *t*-test in Excel (paired-samples *t*-test). Differences were determined to be statistically significant if *P* < 0.05 with 95% confidence level, among measured elements, and between washed and unwashed plants. SPSS version 22 was used, comparing means using one-way ANOVA, to investigate differences among the plant species for the individual elements with significance level of 0.05.

## 3. Results

### 3.1. Limits of Detection (LODs) and Limits of Quantification (LOQs)

The LODs (*μ*g/L) were as follows: Fe 0.34, Cu 0.03, Se 0.07, Zn 0.17, and Mn 0.04. The LOQs (*μ*g/L) were as follows: Fe 1.12, Cu 0.08, Se 0.24, Zn 0.56, and Mn 0.12.

### 3.2. Quality Control

The average recoveries of the CCV for each element in one session after each set of five measurements were as follows: Fe 79.57%, Cu 91.57%, Se 88.20%, Zn 90.50%, and Mn 84.90%. The recoveries of the spiked sample with the five elements were as follows: Fe 125.08%, Cu 83.54%, Se 94.39%, Zn 92.82%, and Mn 99.27%.

### 3.3. Concentrations of the Essential Elements in the Medicinal Plants and Statistical Analysis

The concentrations (*μ*g/g) of the five measured elements in unwashed and washed plant samples are shown in Table 1. Triplicate measurements (*n*=3) for each sample were carried out. In general, washed plants showed lower concentrations than unwashed plants for all elements measured. This indicates that washing removed essential elements from the plants. In some cases, the removal was as much as 80% of the original concentration in the unwashed sample. The reason for washing the plants was to investigate the impact of washing on the level of original concentrations of the essential elements. We can derive from these results that rigorous washing will remove high percentages of essential elements in plants in the filtrate. Therefore, the users who drink the filtrate can ingested the removed percentage, which can be source of the essential elements in such plants. The level of essential elements in filtrate can be calculated as a difference between unwashed and washed plants (Table 2). It is noteworthy that nabipoot showed high levels of Mn, Zn, and Cu in the washed sample, although contribution from water (deionized water) used for washing was negligible. Moreover, only harjal and cafmariam showed the same trend for Cu, and this could be caused by a contamination. However, other plants were not affected by such a trend.

Mahareeb *(Cymbopogon)* showed higher concentrations of the measured elements compared with the other plants. The levels of elements for all medicinal plants were as follows: Fe > Mn > Zn > Cu > Se. The increasing order of the plants based on the concentrations of each individual element as follows:

  Fe: mahareeb > nabipoot > cafmariam > harjal > sheeh
  Cu: mahareeb > nabipoot > harjal > sheeh > cafmariam
  Zn: sheeh > harjal > mahareeb > nabipoot > cafmariam
  Se: nabipoot > harjal > mahareeb > sheeh > cafmariam
  Mn: harjal > mahareeb > nabipoot > sheeh > cafmariam

Thus, mahareeb has a tendency to the highest contents of elements, whereas cafmariam has a tendency to the lowest in most of the cases.

Based on comparison among the five plants, Fe contents in all plants showed significant difference (*P* < 0.05) from each other. Mahareeb reported the highest value, and sheeh had the lowest level. From the statistical analysis for Cu levels among the five plants, mahareeb and nabipoot showed no significant difference (*P* > 0.05), while the other three plants showed significant difference (*P* < 0.05) among each other. Mahareeb reported the highest value, and cafmariam had the lowest level.

Based on the outcome of the statistical analysis among the five plants regarding the Mn content, there were significant differences (*P* < 0.05) among all plants. Harjal reported the highest value for Mn, and cafmariam had the lowest level. Moreover, the statistical analysis regarding Zn levels in the five plants showed no significant difference (*P* > 0.05) between mahareeb and nabipoot. However, the other three plants showed significant difference among each other. Sheeh reported the highest value, and cafmariam had the lowest level. The statistical analysis related to Se levels among the five plants showed significant difference (*P* < 0.05) between mahareeb and nabipoot. No significant difference (*P* > 0.05) was shown among the rest. Nabipoot had the highest value, and cafmariam had the lowest level. A significant difference (*P* < 0.05) was observed in the levels of most measured elements between the washed and unwashed plants.

Washing removed a high percentage of Fe from all plants (33%–65%). High percentages of all elements in the range 19%–80% were removed from mahareeb by washing, compared with all other plants. Nabipoot showed the lowest percentage of elements removed by washing (0%–51%), compared with all other plants.

### 3.4. Calculations of Intake of the Elements in Each Dose of a Plant

Based on personal communications with consumers and retailers, the quantity of each plant used in one dose is as follows: harjal 7.39 g, nabipoot 3.59 g, mahareeb 3.33 g, sheeh 4.49 g, and cafmariam 4.33 g. The quantity of each plant was calculated as an average of few small bunches. We calculated the quantities of each element contained in each dose of the medical plants in this study. The doses shown above for each plant were multiplied by the concentration of each measured element (Table 1), using the following equation: *D* (g) × *C*_elm_ (*μ*g/g), where *D* = amount of one dose in grams (bunch) and *C*_elm_ = measured concentration of each element in the plant in *μ*g/g. The calculated quantities for the elements in the unwashed and washed plants are shown in Table 2. The leachability of elements by using hot water was calculated from infusion. One gram of each plant was boiled in deionized water for more than half an hour and then filtered and made up to 100 mL by adding deionized water. Concentrations of all elements were measured in the filtrate solution. The measured concentrations of essential elements for the medical plants in infusion are included in Table 1. Their ranges were as follows: Fe 1.25–3.41, Mn 1.56–14.77, Zn 1.90–5.77, Se 0.03–0.43, and Cu 0.42–0.998 *µ*g/g. The calculated dosages of exposure (*μ*g/dose) are included in Table 2. These dosages of exposure were compared to the levels prescribed by the provisional maximum tolerable daily intake (PMTDI) by the WHO and European Food Safety Authority (EFSA) as shown in Table 3 [6–8, 21].

## 4. Discussion

The investigated medicinal plants in this study are a source of the essential elements Fe, Cu, Zn, Se, and Mn. Essential elements are important for human health [6]. In general, all the five plants are source of higher level of Fe and lower level of Se.

Previous studies have been conducted to determine the levels of essential elements in medicinal plants. In Saudi Arabia, the element contents in various medicinal plants (different from this study) were already determined [22]. These authors reported the concentrations of essential elements, namely, Se, Mn, Zn, and Cu. Our study also reported the concentrations of these elements (Table 1). Zn (0.5–41.1 *μ*g/g) and Cu (2.2–22.6 *μ*g/g) levels in this study were within the range found in our study; Se (0.2–3.4 *μ*g/g) had a larger range than observed in our study; Mn (2.6–102.9 *μ*g/g) had a similar range to our study. Iron was reported in high levels in all tested medicinal plants in this study as well as in ours. These results showed that all medicinal plants, regardless of their origins, are a source of similar high concentrations of micronutrients, such as iron.

The levels of trace elements, including Cu, Fe, Zn, Mn, and Se, have been determined in Chinese herbal medicines used for relieving heat [23]. The results from their study showed a similar trend as found in our study, in which Fe (87.85–1951.86 *μ*g/g) was present in the highest concentration in all investigated herbs. These results demonstrate that medicinal plants with different origins can show similar trends in the levels of essential elements, which is the trend of many terrestrial plants. It was reported that trace element uptake by plants due to their concentrations in nutrient solutions, which reflect the similar soil availability [24]. The absorption of elements by plants depends on several soil properties and chemical composition, besides highly mobile elements (Cd, Zn, and Mo) and less mobile elements (Cr, Ni, Pb, As, Co, and Cu) [24, 25]. Moreover, the uptake of the heavy metals by plants from the soil is the same as macro- and micronutrients through the root system [25].

The results from a recent study [26] investigating the accumulation of trace elements in two medicinal plants (*Azadirachta indica* and *Ocimum sanctum*) from India is consistent with the trends observed in our study. Additionally, Zn (200.8–254.5 *μ*g/g) > Cu (63.5–129.2 *μ*g/g) followed the same trend as observed for all plant samples in the present study. This similar trend could indicate that different herbal plants in different geographic areas uptake elements in similar ratios.

Another recent study [27] measured the levels of trace elements in plant leaves from five medicinal plants and found the following trend: Fe (2.827–36.39 *μ*g/g) > Mn (0.321–19.63 *μ*g/g) > Zn (0.086–0.146 *μ*g/g) > Cu (0.075–0.091 *μ*g/g), which was the same trend observed for all the medicinal plants in our study. Again, it can be seen that similar proportions of elements are present in plants regardless of the geographic area of origin.

A study [28] in the United Arab Emirates (UAE) measured the concentrations of heavy metals in commonly consumed herbs in the UAE. The results from this study agree with our study. The measured trend in element concentrations was as follows: Fe (81.25–1101.22 *μ*g/g) > Zn (12.65–146.67 *μ*g/g) > Cu (1.44–156.24 *μ*g/g). Again, this trend matches what was found in the present study.

From our previous study [29], surprisingly a trend of decreasing order of the essential elements was as follows: Mn > Zn > Cu in yellow shamma (consisting mostly from pure tobacco). This trend of decreasing order is the same as our results. This may defer that different plants in the same geographic area have a similar trend in uptake of essential elements. Same observations were also reported for toxic elements (As, Cd, Pb, and Al), compared with other studies, in our recent study [20] for the same medicinal plants.

## 5. Conclusion

We conclude from this study that medicinal plants are an important source of essential elements, such as Fe, Mn, Zn, Cu, and Se. Fe and Mn were the elements in the highest concentrations in all plants, with Se having the lowest concentration in all plants. In general, mahareeb contributed to higher levels of the essential elements compared with all other plants, while cafmariam reported the lowest levels of the essential elements. The washing process removed a percentage of the essential elements from all investigated plants. Infusion of all plants was measured, and calculated intake levels of essential elements per dose were below the recommended daily intake levels set by the WHO and EFSA.

## Figures and Tables

**Table 1:** Concentrations (*μ*g/g) of the five essential elements measured in unwashed, washed, and infusion in the five medicinal plants (*n*=3; mean ± SD).

Elements	Mahareeb	Sheeh	Harjal	Nabipoot	Cafmariam
*Unwashed plants (μg/g)*					
Fe	1757.9 ± 2.8	193.4 ± 10.6	399.7 ± 6.7	859.8 ± 0.8	580.4 ± 9.0
Cu	21.8 ± 0.1	18.0 ± 0.1	20.6 ± 0.1	21.7 ± 0.0	11.3 ± 0.1
Zn	17.7 ± 0.2	32.7 ± 0.8	19.7 ± 0.3	17.1 ± 1.2	15.4 ± 0.3
Se	0.3 ± 0.0	0.1 ± 0.0	0.31 ± 0.0	0.92 ± 1.7	0.13 ± 0.0
Mn	121.5 ± 0.9	33.0 ± 0.8	143.7 ± 2.1	77.0 ± 1.6	23.6 ± 0.4
*Washed plants (μg/g)*					
Fe	614.3 ± 12.6	160.1 ± 4.3	248.2 ± 3.5	576.4 ± 5.7	237.3 ± 1.8
Cu	10.0 ± 0.1	17.49 ± 0.0	37.66 ± 0.1	24.0 ± 0.1	52.1 ± 0.2
Zn	14.4 ± 0.3	8.0 ± 0.1	12.8 ± 0.2	21.0 ± 0.2	5.0 ± 0.1
Se	0.1 ± 0.0	0.1 ± 0.0	0.2 ± 0.0	0.6 ± 0.0	0.1 ± 0.0
Mn	98.4 ± 0.1	28.4 ± 0.7	110.6 ± 1.5	94.8 ± 1.6	13.3 ± 0.2
*Measured concentration (μg/g) in infusion of each medical plant after boiling with deionized water*					
Fe	1.4 ± 0.0	2.3 ± 0.0	3.4 ± 0.1	2.3 ± 0.0	1.3 ± 0.4
Cu	0.6 ± 0.0	1.0 ± 0.0	0.7 ± 0.0	0.4 ± 0.0	0.4 ± 0.0
Zn	2.1 ± 0.1	4.3 ± 0.1	5.8 ± 0.1	4.7 ± 0.1	1.9 ± 0.0
Se	0.1 ± 0.0	0.1 ± 0.0	0.1 ± 0.0	0.4 ± 0.0	0.03 ± 0.0
Mn	14.8 ± 0.2	13.0 ± 0.3	16.7 ± 0.2	9.0 ± 0.3	1.6 ± 0.0

**Table 2:** Calculated levels (mg) of essential elements measured in unwashed and washed plants, based on the commonly used quantity of each plant in one dose (g), and calculated dosage of exposure (*μ*g) in infusion.

Elements	Mahareeb	Sheeh	Harjal	Nabipoot	Cafmariam
*Unwashed plants (mg)*					
Fe	5.86	0.72	2.95	3.09	2.52
Cu	0.073	0.081	0.152	0.078	0.049
Zn	0.059	0.147	0.146	0.061	0.067
Se	0.00083	0.00063	0.00229	0.0033	0.00056
Mn	0.405	0.149	1.061	0.276	0.102
*Washed plants (mg)*					
Fe	2.05	8.72	1.83	2.07	1.03
Cu	0.033	0.079	0.278	0.086	0.086
Zn	0.0478	0.0359	0.0945	0.0755	0.0215
Se	0.00017	0.0004	0.00163	0.00226	0.00035
Mn	0.3278	0.1275	0.8172	0.3401	0.0578
*Calculated dosage of exposure (μg) in infusion of each medical plant after boiling with deionized water*					
Fe	4.63	13.39	25.22	8.36	5.40
Cu	1.92	4.48	5.00	1.51	1.83
Zn	7.04	19.47	42.65	16.72	8.23
Se	0.22	0.57	0.68	1.55	0.14
Mn	49.17	58.24	123.15	32.33	6.72

**Table 3:** Provisional maximum tolerable daily intake (PMTDI) of essential elements (Fe, Se, Zn, Mn, and Cu).

Number	Element	PMTDI	Reference
1	Zinc (Zn)	1 mg/kg bw per day (70 mg/day^∗^)	[21]
2	Copper (Cu)	0.5 mg/kg bw per day (35 mg/day^∗^)	[21]
3	Selenium (Se)	70 *μ*g/day for adults^$^	[7]
4	Manganese (Mn)	3 mg/day for adults^$^	[6]
5	Iron (Fe)	11 mg/day^£^	[8]

^∗^Calculated value based on PMTDI, equivalent to mg/day for a 70 kg person; ^$^adequate intake (AI)/day; ^£^opulation reference intake (PRI).
